# Exact *p*-values for pairwise comparison of Friedman rank sums, with application to comparing classifiers

**DOI:** 10.1186/s12859-017-1486-2

**Published:** 2017-01-25

**Authors:** Rob Eisinga, Tom Heskes, Ben Pelzer, Manfred Te Grotenhuis

**Affiliations:** 1 Department of Social Science Research Methods, Radboud University Nijmegen, PO Box 9104, , 6500 HE Nijmegen, The Netherlands; 2 Institute for Computing and Information Sciences, Radboud University Nijmegen, Nijmegen, The Netherlands

**Keywords:** Friedman test, Exact *p*-value, Rank sum difference, Multiple comparison, Nonparametric statistics, Classifier comparison, Machine learning

## Abstract

**Background:**

The Friedman rank sum test is a widely-used nonparametric method in computational biology. In addition to examining the overall null hypothesis of no significant difference among any of the rank sums, it is typically of interest to conduct pairwise comparison tests. Current approaches to such tests rely on large-sample approximations, due to the numerical complexity of computing the exact distribution. These approximate methods lead to inaccurate estimates in the tail of the distribution, which is most relevant for *p*-value calculation.

**Results:**

We propose an efficient, combinatorial exact approach for calculating the probability mass distribution of the rank sum difference statistic for pairwise comparison of Friedman rank sums, and compare exact results with recommended asymptotic approximations. Whereas the chi-squared approximation performs inferiorly to exact computation overall, others, particularly the normal, perform well, except for the extreme tail. Hence exact calculation offers an improvement when small *p*-values occur following multiple testing correction. Exact inference also enhances the identification of significant differences whenever the observed values are close to the approximate critical value. We illustrate the proposed method in the context of biological machine learning, were Friedman rank sum difference tests are commonly used for the comparison of classifiers over multiple datasets.

**Conclusions:**

We provide a computationally fast method to determine the exact *p*-value of the absolute rank sum difference of a pair of Friedman rank sums, making asymptotic tests obsolete. Calculation of exact *p*-values is easy to implement in statistical software and the implementation in R is provided in one of the Additional files and is also available at http://www.ru.nl/publish/pages/726696/friedmanrsd.zip.

**Electronic supplementary material:**

The online version of this article (doi:10.1186/s12859-017-1486-2) contains supplementary material, which is available to authorized users.

## Background

The Friedman [1] rank sum test is a widely-used nonparametric method for the analysis of several related samples in computational biology and other fields. It is used, for example, to compare the performance results of a set of (expression-based) classifiers over multiple datasets, covering case problems, benchmark functions, or performance indicators [2–4]. Some recent examples of the numerous applications of the Friedman test in bioinformatics include [5–17]. The test is supported by many statistical software packages and it is routinely discussed in textbooks on nonparametric statistics [18–23].

The Friedman test procedure is an analysis of variance by ranks, i.e., observed rank scores or rank scores obtained by ordering ordinal or numerical outcomes, that is used when one is not willing to make strong distributional assumptions. A common approach is to present the test as a method for identifying treatment effects of *k* different treatments in a so-called randomized complete block design. This design uses *n* sets, called blocks, of *k* homogeneous units matched on some relevant characteristic, for example patients matched on SNP genotype. The *k* treatments are assigned randomly to the *k* units within each block, with each treatment condition being administered once within a block. The Friedman test is also conducted if the samples concern a repeated measures design. In such design each experimental unit constitutes a block that serves in all treatment conditions. Examples are provided by experiments in which *k* different treatments (classifiers) are compared on a single experimental unit (dataset), or if *k* units (e.g., genes, products, candidates) are ranked in order by each of *n* ‘judges’ (algorithms, panelists). In all these settings the objective is to determine if the *k* populations from which the observations were made are identically distributed.

Applied to classifier comparison, the null hypothesis for the Friedman test is that the performance results of the *k* classifiers over *n* datasets are samples that have been drawn from the same population or, equivalently, from different populations with the same distribution [18]. To examine this hypothesis, the test determines whether the rank sums of the *k* classifiers included in the comparison are significantly different. After applying the omnibus Friedman test and observing that the rank sums are different, the next step is to compare all classifiers against each other or against a baseline classifier (e.g., newly proposed method or best performing algorithm). In doing so, a series of comparisons of rank sums (i.e., rank sum difference tests) is performed, adjusting the significance level using a Bonferroni correction or more powerful approaches to control the familywise Type-I error rate [3, 4].

Preferably one should use the exact null distribution of the rank sum difference statistic in these subsequent analyses. Only if the decision on the null hypothesis is based on the exact distribution is the probability of committing a Type-I error known exactly. However, the exact distribution and the associated true tail probabilities are not yet adequately known. To be sure, tables of exact critical values at standard significance levels (e.g., [18, 21, 22]) and of exact *p*-values [24] are available for small values of *k* and *n*, for which complete enumeration is possible. Also, the lower order moments of the exact sampling distribution have been documented in detail [25], and Stuart [26] proved the conjecture of Whitfield [24] that, on the null hypothesis, the difference between rank sum values is asymptotically normally distributed as *n* tends to infinity. Further, in a recent study Koziol [27] used symbolic computation for finding the distribution of absolute values of differences in rank sums. Apart from these important contributions there is, to the best of our knowledge, no publication available in the probability theory, rank statistics or other literature that addresses the exact distribution of the rank sum difference statistic.

As the null distribution in the general case is unknown and exact computation seemingly intractable, it is generally recommended to apply a large-sample approximation method to test the significance of the pairwise difference in rank sums. It is well known, however, that calculating probabilities using an asymptotic variant of an exact test may lead to inaccurate *p*-values when the sample size *n* is small, as in most applications of the Friedman test, and thereby to a false acceptance or false rejection of the null hypothesis. Furthermore, there are several large-sample tests available with different limiting distributions, and these tests may vary substantially in their results. Consequently, there is little agreement in the nonparametric literature over which approximate method is most appropriate to employ for the comparison of Friedman rank sums [22]. This statement refers both to approximate tests of significance for the comparison of all (_2_^*k*^) = *k*(*k* − 1)/2 pairs of treatments, and to tests for the comparison of *k* − 1 treatments with a single control. Obviously, the utility of the asymptotic tests depends on their accuracy to approximate the exact sampling distribution of the discrete rank sum difference statistic.

The purpose of this note is twofold. The foremost aim is to provide an expression for calculating the exact probability mass function of the pairwise differences in Friedman rank sums. This expression may be employed to quickly calculate the exact *p*-value and associated statistics such as the critical difference value. The calculation does not require a complicated algorithm and as it is easily incorporated into a computer program, there is no longer need to resort to asymptotic *p*-values. We illustrate the exact method in the context of two recently published analyses of the performance of classifiers and data projection methods. The second aim is to examine under what circumstances the exact distribution and the associated exact statistics offer an improvement over traditional approximate methods for Friedman rank sum comparison.

It is important to note at the outset that this article is not concerned with the Friedman test itself. The Friedman test is an over-all test that evaluates the joint distribution of rank sums to examine equality in treatment distributions. Computation of the exact joint distribution under the null is discussed by van de Wiel [28], and an efficient algorithm for computing the exact permutation distribution of the Friedman test statistic is available in StatXact [29]. The present paper offers an over-all exact test for pairwise comparison of Friedman rank sums. The reason is essentially that researchers are usually not only interested in knowing whether any difference exists among treatments, but also in discovering *which* treatments are different from each other, and the Friedman test is not designed for this purpose. Although the type of test dealt with here is not the same as the Friedman test, we will briefly discuss the latter as the procedures have important elements in common, such as the global null hypothesis. Also, we assume in our discussion that the available data are such that it is appropriate to perform simultaneous rank sum tests. Hence, we ignore empirical issues such as insufficient power (too few datasets), selection bias (nonrandom selection of datasets), and like complications that, as noted by Boulesteix *et al*. ([30]; see also [31]), tend to invalidate statistical inference in comparative benchmarking studies of machine learning methods solving real-world problems. In ANOVA, the term ‘treatment’ is used as a common term for the grouping variable for which a response is measured. To accommodate the wide variety of applications of the Friedman test, the more general term ‘group’ is used instead of ‘treatment’ in the remainder of this paper. The term subject is used hereafter to include both objects and individuals.

## Methods

### Friedman data

To perform the Friedman test the observed data are arranged in the form of a complete two-way layout, as in Table 1A, where the *k* rows represent the groups (classifiers) and the *n* columns represent the blocks (datasets).

**Table 1:**
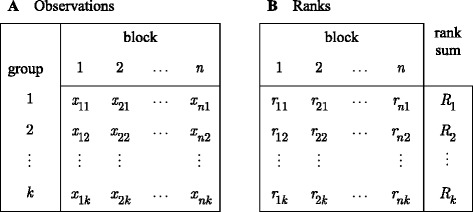
Two-way layout for Friedman test

The data consist of *n* blocks with *k* observations within each block. Observations in different blocks are assumed to be independent. This assumption does not apply to the *k* observations within a block. The test procedure remains valid despite within-block dependencies [32]. The Friedman test statistic is defined on ranked data so unless the original raw data are integer-valued rank scores, the raw data are rank-transformed. The rank entries in Table 1B are obtained by first ordering the raw data {*x*_*ij*_; *i* = 1, …, *n*, *j* = 1, …, *k*} in Table 1A column-wise from least to greatest, within each of the *n* blocks separately and independently, and then to assign the integers 1,…,*k* as the rank scores of the *k* observations within a block. The row sum of the ranks for any group *j* is the rank sum defined as *R*_*j*_ = ∑_*i* = 1_^*n*^ *r*_*ij*_.

### Null hypothesis

The general null hypothesis of the Friedman test is that all the *k* blocked samples, each of size *n*, come from identical but unspecified population distributions. To specify this null hypothesis in more detail, let *X*_*ij*_ denote a random variable with unknown cumulative distribution function *F*_*ij*_, and let *x*_*ij*_ denote the realization of *X*_*ij*_.

The null hypothesis can be defined in two ways, depending on whether blocks are fixed or random [33]. If blocks are fixed, then all the *k* × *n* measurement values are independent. If there are *k* groups randomly assigned to hold *k* unrelated *X*_*ij*_ within each block, as in a randomized complete block design, then the null hypothesis that the *k* groups have identical distributions may be formulated as

*H*_0_ : *F*_*i*1_(*x*) = … = *F*_*ik*_(*x*) = *F*_*i*_(*x*) for each *i* = 1, …, *n*,

where *F*_*i*_(*x*) is the distribution of the observations in the *i*th block [28, 33]. The same hypothesis, but more specific, is obtained if the usual additive model is assumed to have generated the *x*_*ij*_ in the two-way layout [23]. The additive model decomposes the total effect on the measurement value into an overall effect *μ*, block *i* effect *β*_*i*_, and group *j* effect *τ*_*j*_. If the distribution function is denoted *F*_*ij*_(*x*) = *F*(*x* − *μ* − *β*_*i*_ − *τ*_*j*_), the null hypothesis of no differences among the *k* groups may be stated as

$$ {H}_0:\kern0.5em {\tau}_1=\dots ={\tau}_k, $$

and the general alternative hypothesis as

${H}_1:\kern0.5em {\tau}_{j_1}\ne {\tau}_{j_2}$ for at least one (*j*_1_, *j*_2_) pair.

Note that this representation also asserts that the underlying distribution functions *F*_*i*1_(*x*), …, *F*_*ik*_(*x*) within block *i* are the same, i.e., that *F*_*i*1_(*x*) = … = *F*_*ik*_(*x*) = *F*_*i*_(*x*), for each fixed *i* = 1, …, *n*.

If blocks are random, measurements from the same random block will be positively correlated. For example, if a single subject forms a block and *k* observations are made on the subject, possibly in randomized order, the within-block observations are dependent. Such dependency occurs in a repeated measures design where *n* subjects are observed and each subject is tested under *k* conditions. Denote the joint distribution function of observations within block *i* by *F*_*i*_(*x*_1_, …, *x*_*k*_). Then the null hypothesis of no differences among the *k* groups is the hypothesis of exchangeability of the random variables *X*_*i*1_, …, *X*_*ik*_ [28, 34], formulated as

*H*_0_ : *F*_*i*_(*x*_1_, …, *x*_*k*_) = *F*_*i*_(*x*_*σ*(1)_, …, *x*_*σ*(*k*)_) for *i* = 1, …, *n*,

where *σ*(1), …, *σ*(*k*) denotes any permutation of 1, …, *k*. The model underlying this hypothesis is that the random variables *X*_*ij*_ have an exchangeable distribution. This is a suitable model for repeated measures, where it is not appropriate to assume independence within a block [32, 33]. We also note that this formulation of the null hypothesis and the one for fixed blocks are consistent against the same alternative, namely the negation of *H*_0_. For a detailed discussion of this matter, see [35].

Whether blocks are fixed or random, if the null hypothesis is true, then all the permutations of 1, …, *k* are equally likely. There are *k* ! possible ways to assign *k* rank scores to the *k* groups within each block and all these intra-block permutations are equiprobable under *H*_0_. As the same permutation argument applies to each of the *n* independent blocks, there are (*k* !)^*n*^ equally likely rank configurations of the rank scores *r*_*ij*_ in the two-way layout [23]. Each of these permutations has a probability of (*k* !)^− *n*^ of being realized. This feature is used to evaluate the null distribution of the rank sums *R*_*j*_, by enumerating all the permutations of the two-way layout of ranks.

### Friedman test statistic

Under the Friedman null hypothesis, the expected row sum of ranks for each group equals *n*(*k* + 1)/2. The Friedman test statistic

$$ {X}_r^2=\frac{12}{nk\left( k+1\right)}{\displaystyle \sum_{j=1}^k{\left\{{R}_j- n\left( k+1\right)/2\right\}}^2} $$

sums the squared deviations of the observed rank sums for each group, *R*_*j*_, from the common expected value for each group, *n*(*k* + 1)/2, under the assumption that the *k* group distributions are identical. For small values of *k* and *n*, the exact distribution of *X*_*r*_^2^ has been tabled, for example, by Friedman [1]. An algorithm for computing the exact joint distribution of the Friedman rank sums under the null is discussed in [28]. For the special case of two paired samples, see [36].

Calculating the test statistic using the null distribution of the (*k* !)^*n*^ possible permutations is time consuming if *k* is large. However, Friedman [1] showed that as *n* tends to infinity, *X*_*r*_^2^ converges in distribution to *χ*_*df* = *k* − 1_^2^, a chi-squared random variable with *k* − 1 degrees of freedom. This result is used in the asymptotic Friedman test. The Friedman test rejects *H*_0_ at a pre-specified significance level *α* when the test statistic *X*_*r*_^2^ exceeds the 100(1 − *α*)*th* percentile of the limiting chi-squared distribution of *X*_*r*_^2^ with *k* − 1 degrees of freedom [1]. The test statistic needs to be adjusted if there are tied ranks within blocks [22, 23]. Also, various modifications of the Friedman test have been proposed, for example the *F* distribution as an alternative to the chi-squared distribution [37], as well as generalizations, such as the Skillings-Mack [38] test statistic for use in the presence of missing data. These and various other adjustments and nonparametric competitors to the Friedman test (e.g., Kruskal-Wallis, Quade, Friedman aligned ranks test) are not discussed here (see [4, 22, 23]).

### Pairwise comparison tests and approximate critical difference

Frequently, researchers are not only interested in testing the global hypothesis of the equality of groups but also, or even more so, in inference on the equality of equality of pairs of groups. Further, even if one is mainly interested in *H*_0_ and the hypothesis is rejected, a follow-up analysis may be conducted to determine possible reasons for the rejection. Such analysis may disclose group differences, but it might also reveal that none of the pairs is significantly different, despite a globally significant test result.

To address these issues it is expedient to test hypotheses of equality for pairs of groups using simultaneous comparison tests. These multiple comparison procedures may involve, in 1 × *N* (or *many-one*) comparisons, testing *k* − 1 hypotheses of equality of all non-control groups against the study control or, in *N* × *N* (*all-pairs*) comparisons, considering *k*(*k* − 1)/2 hypotheses of equality between all pairs of groups. For both types of comparisons, large-sample approximate tests have been designed. They are derived for the situation where *n*, the number of blocks (i.e., ‘sample size’), is large.

Table 2 displays the critical difference (*CD*) approximate tests for 1 × *N* and *N* × *N* comparisons of Friedman rank sums, as recommended in highly-cited monographs and papers and popular textbooks on nonparametric statistics. The critical difference is the minimum required difference in rank sums for a pair of groups to differ at the pre-specified alpha level of significance. It is to note that in many publications the *CD* statistic is calculated using the difference in rank sum averages, i.e., *R*_*j*_/*n*, rather than rank sums. The results are identical, since each group has *n* observations, if the test statistic formulas are modified appropriately.

**Table 2:** Recommended critical difference (*CD*) approximate tests for 1 × *N* and *N* × *N* comparisons of Friedman rank sums

Comparison	Critical difference	Reference
1 × *N*	$C{D}_N={z}_{\alpha /{c}_1}\sqrt{nk\left( k+1\right)/6},\kern0.75em {c}_1= k-1$	Demšar [2]
	$C{D}_M={m}_{\alpha, df= k-1,\rho ={\scriptscriptstyle \frac{1}{2}}}\sqrt{nk\left( k+1\right)/6}$	Siegel and Castellan [18], Nemenyi [39], Miller [25], Hollander et al. [23], Zarr [20]
*N* × *N*	$C{D}_N={z}_{{\scriptscriptstyle \frac{1}{2}}\alpha /{c}_2}\sqrt{nk\left( k+1\right)/6},\kern0.5em {c}_2= k\left( k-1\right)/2$	Siegel and Castellan [18], Gibbons and Chakraborti [21], Daniel [19], Hettmansperger [33], Sheskin [22]
	$$ \begin{array}{l} C{D}_Q = {q}_{\alpha, df= k,\infty}\sqrt{nk\left( k+1\right)/12}=\\ {}\kern3.25em \frac{q_{\alpha, df= k,\infty }}{\sqrt{2}}\sqrt{nk\left( k+1\right)/6}\end{array} $$	Nemenyi [39], Miller [25], Hollander et al. [23], Zarr [20], Desu and Raghavarao [40], Demšar [2]
	$C{D}_{\chi^2}=\sqrt{\chi_{\alpha, df= k-1}^2}\sqrt{nk\left( k+1\right)/6}$	Miller [25], Bortz et al. [41], Wike [42]

*Note*: The constant ${m}_{\alpha, df= k-1,\rho ={\scriptscriptstyle \frac{1}{2}}}$ is the upper *α*th percentile point for the distribution of the maximum of *k* − 1 equally correlated (*ρ*=.5) unit normal *N*(0, 1) random variables. The constant *q*_*α*,*df* = *k*,∞_ is the upper *α*th percentile point of the Studentized range (*q*) distribution with (*k*, ∞) degrees of freedom. The references in the right-most column are ordered by year of publication (of first edition).

When the null hypothesis of equidistribution of ranks in *n* independent rankings is true, and the condition of a large sample size is met, the differences in rank sums are approximately normally distributed [26]. Let *d* = *R*_*i*_ − *R*_*j*_, with *i* ≠ *j*, be the rank sum difference among a pair of groups *i* and *j*. The support of rank sum difference *d* is the closure [−*n*(*k* − 1), *n*(*k* − 1)]. Under the null hypothesis, the expected value *E*(*d*) = 0 and the variance Var(*d*) = *nk*(*k* + 1)/6 [18, 23, 25]. As the distribution of *d* is symmetric around *E*(*d*) = 0, the skewness is zero, as are all odd order moments. The kurtosis coefficient, derived by Whitfield [24] as

$$ \mathrm{Kurt}(d)=3-\frac{3}{5 n}-\frac{12}{5 n k}-\frac{6}{5 n k\left( k+1\right)}, $$

is less than 3 (i.e., negative excess kurtosis), implying that the discrete rank sum difference distribution has thinner tails than the normal. Notice, however, that the kurtosis tends to 3 with increasing *n*, thus a normal approximation is reasonable. This implies that *d* has an asymptotic *N*(0, Var(*d*)) distribution and that the normal deviate $d/\sqrt{\mathrm{Var}(d)}$ is asymptotically *N*(0, 1).

As can be seen in Table 2, the normal approximate test is recommended by various authors when all groups are to be compared against each other pairwise. It is also discussed by Demšar [2] as a test statistic to be employed when all groups are compared with a single control. Note that the normal test procedures control the familywise Type-I error rate by dividing the overall level of significance *α* by the number of comparisons performed (i.e., *c*_1_ in 1 × *N*, and *c*_2_ in *N* × *N* comparisons). There are more powerful competitors to this Bonferroni-type correction available, such as the Holm, Hochberg, and Hommel procedures. These methods to control the overall false positive error rate are not elaborated in this paper. For a tutorial in the realm of classifier comparison, see Derrac et al. [4].

In addition to the ordinary normal approximation, simultaneous tests have been proposed that exploit the covariance structure of the distribution of the values of differences in rank sums. Whereas the *n* rankings are mutually independent under *H*_0_, the rank sums and the rank sum differences are dependent and correlated as well. The correlation among the rank sum differences depends on the rank sums involved. Specifically, as reported by Miller [25], when the null hypothesis is true

$$ \mathrm{C}\mathrm{o}\mathrm{r}\left({R}_i-{R}_j,{R}_i-{R}_l\right)={\scriptscriptstyle \frac{1}{2}}\kern2.25em  i\ne j\ne l $$

$$ \mathrm{C}\mathrm{o}\mathrm{r}\left({R}_i-{R}_j,{R}_l-{R}_m\right)=0\kern2.25em  i\ne j\ne l\ne m. $$

Hence the correlation is zero for pairs of rank sum differences with no group in common, and 0.5 for pairs of differences with one group in common to both differences. The number of correlated pairs decreases as *k* increases. For a study involving *k* groups, the proportion of correlated pairs equals 4/(*k* + 1) [43]. Hence when *k* = 7, for example, 50% of the pairs are correlated, but when *k* = 79 only 5% are correlated.

As noted in various studies (e.g., [23, 25, 39]), for 1 × *N* comparisons this correlation structure implies that, when *H*_0_ is true and *n* tends to infinity, the distribution of the differences between the *k* − 1 group rank sums and the control rank sum coincides with an asymptotic (*k* − 1) -variate normal distribution with zero means. The critical difference value can therefore be approximated by the test statistic labeled *CD*_*M*_ in Table 2, where the constant ${m}_{\alpha, df= k-1,\rho ={\scriptscriptstyle \frac{1}{2}}}$ is the upper *α*th percentile point for the distribution of the maximum value of (*k* − 1) equally correlated *N*(0,1) random variables with common correlation $\rho ={\scriptscriptstyle \frac{1}{2}}.$ The procedure has an asymptotic familywise error rate equal to *α* [23, 25].

For *N* × *N* comparisons, it means that the covariance of the rank sum differences equals the covariance of the differences between *k* independent random variables with zero means and variances *nk*(*k* + 1)/12. Thus, the asymptotic distribution of $max\left\{\left|{R}_i-{R}_j\right|\right\}/\sqrt{nk\left( k+1\right)/12}$ coincides with the distribution of the range (*Q*_*k*,∞_) of *k* independent *N*(0, 1) random variables. The associated test statistic is *CD*_*Q*_, where the constant *q*_*α*,*df* = *k*,∞_ is the upper *α*th percentile point of the Studentized range (*q*) distribution with (*k*, ∞) degrees of freedom [23, 25, 39]. Again, as the test considers the absolute difference of all *k* groups simultaneously, the asymptotic familywise error rate equals *α* [23, 25].

The Friedman statistic test itself gives rise to the simultaneous test mentioned in the bottom row of Table 2. The null hypothesis is accepted if the difference in rank sums fails to exceed the critical value $C{D}_{\chi^2}.$ This asymptotic chi-squared approximation is recommended in some popular textbooks, although Miller [25] has argued that the probability statement is not the sharpest of tests.

### Statistical power and alternative tests

Note that the *CD* test statistics presented in Table 2 do not require information about the within-block ranks as determined in the experiment. Rather, the simultaneous rank tests all assume that within each block each observation is equally likely to have any available rank. When this is true, the quantity (*k* + 1)(*k* − 1)/12 is the variance of the within-block rankings and *nk*(*k* + 1)/6 the variance of the difference between any two rank sums [25]. Hence the null distribution of *d* in the population has zero mean and *known* standard deviation. This is the precise reason why the normal approximate tests use the *z*-score as test statistic. However, it is important to emphasize in this context that the square root of *nk*(*k* + 1)/6 is the standard deviation of *d* when the overall null hypothesis is true, but not when it is false. It holds, similar to *p*-values, only in a particular model, i.e. *H*_0_; a model that may or may not be true. If the null hypothesis is false, the quantity *nk*(*k* + 1)/6 is typically an over-estimate of the variance, and this causes simultaneous tests, approximate and exact, to lose power.

There are pairwise comparison tests for Friedman rank sums available that are computed on the observed rank scores rather than the rank sums. These tests, such as the Rosenthal-Ferguson test [44] and the popular Conover test [45, 46], use the *t*-score as test statistic. The pairwise *t*-tests are often more powerful than the simultaneous tests discussed above, however, there are also drawbacks. In brief, the Rosenthal-Ferguson test uses the observed variances and covariance of the rank scores of each individual pair of groups, to obtain a standard error of *d* for the test of significance of the pairwise rank sum difference. This standard error is valid whether the null hypothesis of no pairwise difference is true or not. However, next to the formal constraint of the test that *n* should be larger than *k* + 1, the variance of *d* may be estimated poorly, as there are typically few degrees of freedom available for (co-)variance estimation in small-sample Friedman test applications. Moreover, the observed (co-)variances are different for each pair of groups. Consequently, it does not follow from the significance of a difference of a given rank sum A from another rank sum B, that a third rank sum C, more different from A than B is, would also be significantly different. This is an unpleasant feature of the test.

The Conover test estimates the standard deviation of *d* by computing a pooled standard error from the (co-)variances of the observed rank scores of all groups, thus increasing statistical power. The method is similar to Fisher’s protected Least Significant Difference (LSD) test, applied to rank scores. In this methodology, no adjustment for multiple testing is made to the *p*-values to preserve the familywise error rate at the nominal level of significance. Rather, the test is protected in the sense that no pairwise comparisons are performed unless the overall test statistic is significant. As in the Fisher protected LSD procedure, the Conover test has the property of incorporating the observed *F*-value of the overall test into the inferential decision process. However, in contrast to the Fisher protected LSD, which uses the observed *F*-value only in a 0–1 (‘go/no go’) manner, the Conover test uses the *F*-value in a smooth manner when computing the LSD. That is, it has the unusual characteristic that the larger the overall test statistic, the smaller the least significant difference threshold is for declaring a rank sum difference to be significant. The Duncan-Waller test [47] has this same characteristic, but this test advocates a Bayesian approach to multiple comparisons with Bayes LSD. As the comparison tests in the second stage are conditional on the result of the first stage, the nominal alpha level used in the pairwise Conover test has no real probabilistic meaning in the frequentist sense. As noted by Conover and Iman ([48]: 2), “Since the *α* level of the second-stage test is usually not known, it is no longer a hypothesis test in the usual sense but rather merely a convenient yardstick for separating some treatments from others.”

### Exact distribution and fast *p*-value calculation

We present an exact test for simultaneous pairwise comparison of Friedman rank sums. The exact null distribution is determined using the probability generating function method. Generating functions provide an elegant way to obtain probability or frequency distributions of distribution-free test statistics [27, 28]. Application of the generating function method gives rise to the following theorem, the proof of which is in Additional file 1.

**Theorem 1** *For n mutually independent integer-valued rankings, each with equally likely rank scores ranging from 1 to k, the exact probability to obtain pairwise difference d for any two rank sums equals*

$$ P\left( D= d; k, n\right)={\left\{ k\left( k-1\right)\right\}}^{- n} W\left( D= d; k, n\right), $$

*where*

$$ W\left( D= d; k, n\right)={\left\{ k\left( k-1\right)\right\}}^n{\displaystyle \sum_{h=0}^n\left(\begin{array}{c}\hfill n\hfill \\ {}\hfill h\hfill \end{array}\right)}\ \frac{1}{k^h{\left(1- k\right)}^n}{\displaystyle \sum_{i=0}^h{\displaystyle \sum_{j=0}^h{\left(-1\right)}^{\left( j- i\right)}}}\left(\begin{array}{c}\hfill h\hfill \\ {}\hfill i\hfill \end{array}\right)\left(\begin{array}{c}\hfill h\hfill \\ {}\hfill j\hfill \end{array}\right)\left(\begin{array}{c}\hfill k\left( j- i\right)- d+ h-1\hfill \\ {}\hfill k\left( j- i\right)- d- h\hfill \end{array}\right) $$

*is the number of distinct ways a rank sum difference of d can arise, with d having support on d* = [−*n*(*k* − 1), *n*(*k* − 1)].

Additional file 1 also offers a closed-form expression for the exact *p*-value of *d.* [49−51] The *p*-value is defined as the probability of obtaining a result at least as extreme as the one observed, given that the null hypothesis is true. It is obtained as the sum of the probabilities of all possible *d*, for the same *k* and *n,* that are as likely or less likely than the observed value of *d* under the null. The exact *p*-value is denoted *P*(*D* ≥ *d*; *k*, *n*), and it is computed using the expression

$$ \begin{array}{l} P\left( D\ge d; k, n\right)={\displaystyle \sum_{h=0}^n\left(\begin{array}{c}\hfill n\hfill \\ {}\hfill h\hfill \end{array}\right)}\ \frac{1}{k^h{\left(1- k\right)}^n}{\displaystyle \sum_{i=0}^h{\displaystyle \sum_{j=0}^h{\left(-1\right)}^{\left( j- i\right)}}}\left(\begin{array}{c}\hfill h\hfill \\ {}\hfill i\hfill \end{array}\right)\left(\begin{array}{c}\hfill h\hfill \\ {}\hfill j\hfill \end{array}\right)\left(\begin{array}{c}\hfill k\left( j- i\right)- d+ h\hfill \\ {}\hfill k\left( j- i\right)- d- h\hfill \end{array}\right),\\ {}\kern27.5em  d=- n\left( k-1\right),\dots, n\left( k-1\right).\end{array} $$

Calculating the exact *p*-value with this triple summation expression provides a speed-up of orders of magnitude over complete enumeration of all possible outcomes and their probabilities by a brute-force permutation approach. For larger values of *n*, however, exact calculation is somewhat time-consuming and to extend the practical range for performing exact tests, it is desirable to compute the *p*-value more efficiently.

Also, because in practice multiple comparison tests are concerned with absolute differences, it is expedient to compute the cumulative probability of the absolute value of differences in rank sums. As the number of mass points of the symmetric distribution of *d* is an integer of the form 2*n*(*k* − 1) + 1, the distribution has an odd number of probabilities. This implies that, as the probability mass function of *d* is symmetric around zero, the probability mass to the left of *d* = 0 may be folded over, resulting in a folded distribution of non-negative *d*. Consequently, the one-sided *p*-value of non-negative *d* in the range *d* = 1, …, *n*(*k* − 1) may be obtained as the sum of the two one-sided *p*-values of the symmetric distribution with support *d* = [−*n*(*k* − 1), *n*(*k* − 1)]. As doubling the one-sided *p*-value leads to a *p*-value for *d* = 0 that exceeds unity, the *p*-value for *d* = 0 (only) is computed as *P*(*D* ≥ 0; *k*, *n*) = *P*(*D* = 0) + *P*(*D* ≥ 1), and this is exactly equal to 1.

To accelerate computation, we transform the double summation over the indices *i* and *j* in the expression for *P*(*D* ≥ *d*; *k*, *n*) to a summation over a single index, *s* say, using Theorem 2. The proof is given in Additional file 2.

**Theorem 2** *For nonnegative integers d and k*

$$ {\displaystyle \sum_{i=0}^h{\displaystyle \sum_{j=0}^h{\left(-1\right)}^{\left( j- i\right)}}}\left(\begin{array}{c}\hfill h\hfill \\ {}\hfill i\hfill \end{array}\right)\left(\begin{array}{c}\hfill h\hfill \\ {}\hfill j\hfill \end{array}\right)\left(\begin{array}{c}\hfill k\left( j- i\right)- d+ h\hfill \\ {}\hfill k\left( j- i\right)- d- h\hfill \end{array}\right)={\displaystyle \sum_{s=0}^h{\left(-1\right)}^s}\left(\begin{array}{c}\hfill 2 h\hfill \\ {}\hfill h+ s\hfill \end{array}\right)\left(\begin{array}{c}\hfill k s- d+ h\hfill \\ {}\hfill k s- d- h\hfill \end{array}\right). $$

This reduction to a singly-sum function implies that the *p*-value can alternatively be calculated from the much simpler expression

$$ P\left( D\ge\ \left| d\right|; k, n\right)=\left\{\begin{array}{c}\hfill 2\ {\displaystyle \sum_{h=0}^n\left(\begin{array}{c}\hfill n\hfill \\ {}\hfill h\hfill \end{array}\right)}\frac{1}{k^h{\left(1- k\right)}^n}{\displaystyle \sum_{s=0}^h{\left(-1\right)}^s\left(\begin{array}{c}\hfill 2 h\hfill \\ {}\hfill h+ s\hfill \end{array}\right)\left(\begin{array}{c}\hfill ks- d+ h\hfill \\ {}\hfill ks- d- h\hfill \end{array}\right)}, \kern1.8em  d=1,\dots, n\left( k-1\right)\hfill \\ {}1\kern22.5em  d=0,\kern3em \end{array}\right. $$

and*,* as we will show, even for larger values of *n* in a computationally fast manner.

### Software implementation

Although the two expressions for the exact *p*-value are mathematically correct, straightforward computation may produce calculation errors. Even for moderate values of *n* (20 or so), the binomial coefficient that has *d* in the indices may become extremely large and storing these numbers for subsequent multiplication creates numerical overflow due to the precision limitation of fixed-precision arithmetic. One way to address this failure is to use a recurrence relation that satisfies the generating function [53, 54]. The recursions we examined were all computationally expensive to run, however, except for small values of *n* and/or *k*. A faster way to compute the exact *p*-value correctly is to use arbitrary-precision arithmetic computation to deal with numbers that can be of arbitrary large size, limited only by the available computer memory.

The calculation of the *p*-value of the absolute rank sum difference *d* given *k* and *n* is implemented in R [55]. The R code, which requires the package Rmpfr [56] for high precision arithmetic to be installed, is in Additional file 3. The script labeled *pexactfrsd* computes the exact *p*-value *P*(*D* ≥ |*d*|), and additionally affords the possibility to compute the probability *P*(*D* = |*d*|), and the (cumulative) number of compositions of *d* (i.e., *W*(*D* = |*d*|) and *W*(*D* ≥ |*d*|)). The R code and potential future updates are also available at http://www.ru.nl/publish/pages/726696/friedmanrsd.zip.

To illustrate the derivations, Additional file 4 offers a small-sized numerical example (*k* = 3, *n* = 2), and Additional file 5 tabulates the number of compositions of *d* for combinations of *k* = *n* = 2,…,6, for inclusion in the OEIS [52]. As can be seen in Additional file 5, for small values of *n* the unfolded, symmetric distribution of *d* is bimodal, with modes at + 1 and − 1 [24]. This feature rapidly disappears as *n* increases, specifically, for *k* > 2 at *n* ≥ 6.

Hereafter, unless otherwise stated, we will consider the value of rank sum difference *d* to be either zero or positive, ranging from 0 to *n*(*k* − 1), and thus drop the absolute value symbol around *d*.

### Incomplete rankings

Because the *n* rankings {1,2,…,*k*} are mutually independent, we may divide them into two (or more), equal or unequal sized parts, labeled (*D*_1_; *k*, *n*_1_) and (*D*_2_; *k*, *n*_2_), with ∑_*t* = 1_^2^ *D*_*t*_ = *D*, and *D*_*t*_ denoting the differences in rank sums of the two parts. The exact *p*-value can be obtained using

$$ P\left( D\ge d; k, n\right)= P\left( D\ge d; k,{n}_1,{n}_2\right)={\displaystyle \sum_{i=-{n}_1\left( k-1\right)}^{n_1\left( k-1\right)} P\left({D}_1= i; k,{n}_1\right)}\times P\left({D}_2\ge \left( d- i\right); k,{n}_2\right), $$

where – as indicated by the summation’s lower bound – calculation is performed using the *p*-value expression that allows for negative *d*. A unique and useful property of the exact method, which is not shared by the approximate methods discussed, is that it is easy to calculate *p*-value probabilities for designs with unequal block sizes *k*; e.g., designs in which *n*_1_ has ranks {1, 2, …, *k*_1_}, and *n*_2_ ranks {1, 2, …, *k*_2_}, with *k*_1_ ≠ *k*_2_. A general expression for calculating the exact *p*-value in incomplete designs with *j* unequal sized parts is

$$ \begin{array}{l} P\left( D\ge d;{k}_1,{n}_1,{k}_2,{n}_2,\cdots, {k}_j,{n}_j\right)={\displaystyle \sum_{i_1=-{n}_1\left({k}_1-1\right)}^{n_1\left({k}_1-1\right)}{\displaystyle \sum_{i_2=-{n}_2\left({k}_2-1\right)}^{n_2\left({k}_2-1\right)}\cdots {\displaystyle \sum_{i_{j-1}=-{n}_{j-1}\left({k}_{j-1}-1\right)}^{n_{j-1}\left({k}_{j-1}-1\right)}} P\left({D}_1={i}_1;{k}_1,{n}_1\right) \times }}\ \\ {}\kern4.25em \\ {}\kern4em  P\left({D}_2={i}_2;{k}_2,{n}_2\right)\times \cdots \times P\left({D}_{j-1}={i}_{j-1};{k}_{j-1},{n}_{j-1}\right)\times P\left({D}_j\ge \left( d-{i}_1-{i}_2\cdots -{i}_{j-1}\right);{k}_j,{n}_j\right),\end{array} $$

where ∑_*t* = 1_^*j*^ *D*_*t*_ = *D*, and an example in which *n* is subdivided into three parts, each with a unique value of *k* (*k*_1_, *k*_2_, *k*_3_), is

$$ \begin{array}{l} P\left( D\ge d;{k}_1,{n}_1,{k}_2,{n}_2,{k}_3,{n}_3\right)={\displaystyle \sum_{i=-{n}_1\left({k}_1-1\right)}^{n_1\left({k}_1-1\right)}{\displaystyle \sum_{j=-{n}_2\left({k}_2-1\right)}^{n_2\left({k}_2-1\right)} P\left({D}_1= i;{k}_1,{n}_1\right) \times }}\\ {}\\ {}\kern13.5em  P\left({D}_2= j;{k}_2,{n}_2\right)\times P\left({D}_3\ge \left( d- i- j\right);{k}_3,{n}_3\right).\end{array} $$

Although the sum functions slow down calculation, this unique feature of exact *p*-value computation enables one to conduct valid simultaneous significance tests whenever some within-block ranks are missing by design. Such tests would be hard to accomplish using one of the large-sample approximation methods. An empirical example will be given in the Application section.

### Exact and mid *p*-values

As pairwise differences with support on *d* = [−*n*(*k* − 1), *n*(*k* − 1)] are symmetrically distributed around zero under *H*_0_, doubling one-sided *p*-value is the most natural and popular choice for an ordinary exact test. A test using exact *p*-value guarantees that the probability of committing a Type-I error does not exceed the nominal level of significance. However, as the Type-I error rate is always below the nominal level, a significance test with exact *p*-value is a conservative approach to testing, especially if the test involves a highly discrete distribution [57]. The mid *p*-value, commonly defined as half the probability of an observed statistic plus the probability of more extreme values, i.e.,

$$ {P}_{\mathrm{mid}}\left( D\ge d; k, n\right)={\scriptscriptstyle \frac{1}{2}} P\left( D= d\right)+ P\left( D> d\right), $$

ameliorates this problem. The mid *p*-value is always closer to the nominal level than the exact *p*-value, at the expense of occasionally exceeding the nominal size.

### Tied rankings

The mid *p*-value may also be used to handle tied rankings. When ties occur within blocks, the midrank (i.e., average of the ranks) is commonly assigned to each tied value. If, as a result of tied ranks, the observed rank sum difference is an integer value *d* plus 0.5, the *p*-value may be obtained as the average of the exact *p*-values of the adjacent integers *d* and *d* + 1, i.e., ${\scriptscriptstyle \frac{1}{2}}\left[ P\left( D\ge d\right)+ P\left( D\ge\ d+1\right)\right],$ and this is equivalent to the mid *p*-value. It is to note that the resulting probability is not exactly valid. Exact *p*-values represent exact frequency probabilities of certain events, and mid *p*-values have no such frequency interpretation. It may be argued, however, that this interpretational disadvantage is of little practical concern and that using mid *p*-values is an almost exact frequency approach. For a discussion of other treatments of ties in rank tests, see [21].

## Results and discussion

### Time performance

**Fig. 1:**
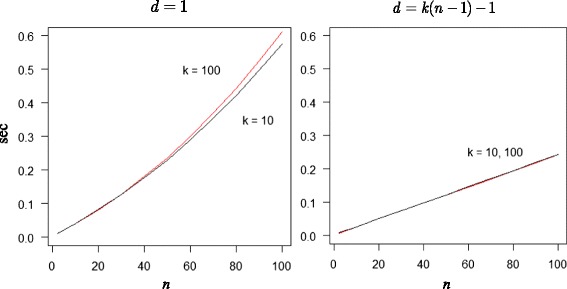
Computational time. Time (in seconds) for calculating the exact *p*-value of *d* = 1 and *d* = *k*(*n* − 1) − 1, for *n* = 2, …, 100 and *k* = 10 (*black line*) and *k* = 100 (*red line*)

The R program computes the exact *p*-value *P*(*D* ≥ *d*; *k*, *n*) at a fast speed. It takes about half a second, for example, to calculate the exact *p*-value for the rather demanding problem *d* = *k* = *n* = 100, on a HP desktop computer using the interpreted R language running under Windows 7 with an Intel Core i7 processor at 2.9GHz. To examine the effects of *d*, *k* and *n* on the algorithm’s runtime, we measured the time it takes to calculate the exact *p*-value of *d* = 1 and *d* = *n*(*k* − 1) − 1, for *n* = 2, …, 100, and *k* = 10 and *k* = 100. The two support values next to the endpoints of the distribution were taken as the *p*-values of the lower and upper support boundaries can be trivially obtained as 1 and 2{*k*(*k* − 1)}^− *n*^, respectively. The computation time (in seconds) is shown in Fig. 1.

The figure indicates that running time is no limitation when it comes to calculating the exact *p*-value, even for larger problems. Computation time is moderately affected by the magnitude of the computed *p*-value. The smaller the *p*-value is, the faster the computation speed. For rank sum difference *d* = 1 running time increases polynomially (of maximum order 3) with increasing *n*, and for *d* = *n*(*k* − 1) − 1 it increases virtually linearly. Also, for *d* = 1, the minor runtime difference between *k* = 10 and *k* = 100 increases slowly with increase in value of *n*. For *d* = *n*(*k* − 1) − 1 the time to do the calculation is essentially the same for *k* = 10 as for *k* = 100. In sum, these timing results show that the exact method admits an algorithm that is fast for all *k* and *n* values typically encountered in empirical studies testing differences in Friedman rank sums, such as those comparing classifiers. This quality makes the algorithm for exact calculation appealing, compared to alternative asymptotic approximations. Indeed, the algorithm is (considerably) faster than the one used here for evaluating the multivariate normal-approximate critical difference (*CD*_*M*_).

### Exact distribution examples

**Fig. 2:**
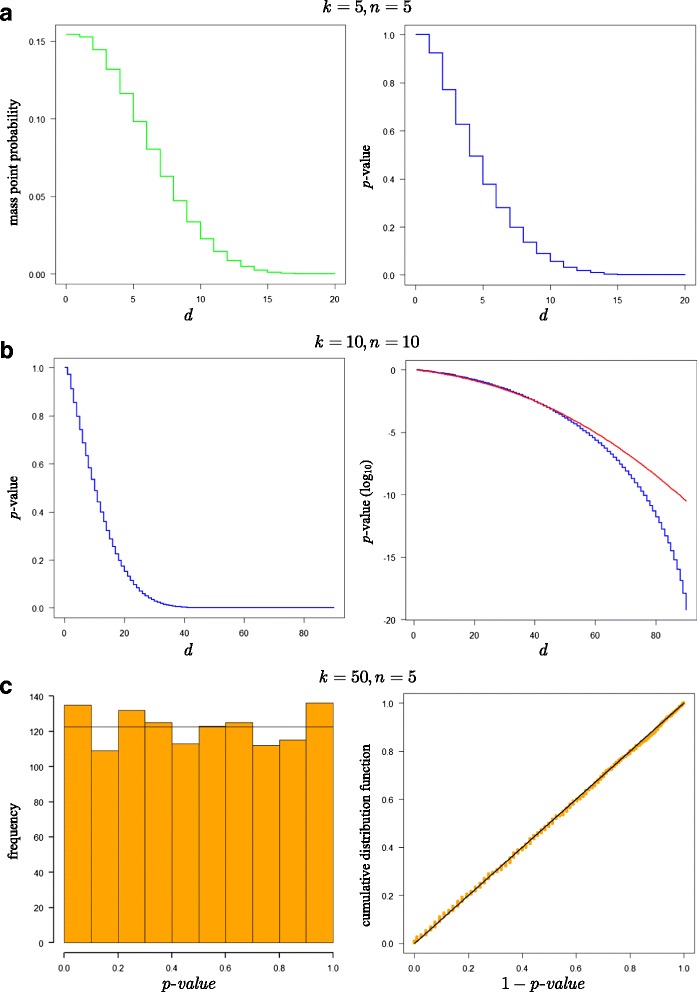
Distribution of exact mass point probabilities and exact *p*-values. **a** Exact mass point probabilities, and exact *p*-values, for *k* = *n* = 5. (**b**) Exact *p*-values, and log10-transformed exact (*blue line*) and normal approximate *p*-values (*red line*), for *k* = *n* = 10. (**c**) Histogram of simulated *p*-values under the overall null hypothesis with expected null frequency superimposed, and cumulative distribution function of the simulated 1 − *p*-values with diagonal line overlay, for *k* = 50, *n* = 5.

We present some examples to illustrate the frequency probability distribution of rank sum difference *d*. The left panel of Fig. 2a displays the mass point probabilities *P*(*D* = *d*; *k*, *n*) for *k* = 5 and *n* = 5, over the entire support interval *d* = [0, 20]. The right panel shows the exact *p*-values *P*(*D* ≥ *d*; *k*, *n*) for *k* = *n* = 5, i.e., the tail-probability at and beyond the value of *d*. The steps in the (cumulative) probability distributions are due to the discreteness of *d*, implying that events are concentrated at a few mass points. To adjust the *p*-values for discreteness, one might opt to obtain mid *p*-values. The mid *p*-value is less than the exact *p*-value by half the mass point probability of the observed result, and it behaves more like the *p*-value for a test statistic with a continuous distribution.

The jumps at the steps decrease with increase in value of *k* and/or *n*. To exemplify this point, the left panel of Fig. 2b displays the less discrete *p*-value distribution for *k* = *n* = 10. The powerful benefit of exact calculation is shown in the right panel of the same figure. The graph displays the log_10_-transformed *p*-values obtained by exact calculation, with the cumulative normal density superimposed. As can be seen, the continuous normal is imperfect for estimating probabilities in the long right tail, where *d* values are large and *p*-values are small. Note that the error increases as the *p*-values decline. Compared to exact calculation, the cumulative normal is overly conservative in that it tends to over-predict the true *p*-value and thus understate the evidence against the null.

For continuous test statistics, *p*-values are known to be uniformly distributed over the interval [0,1] when the null hypothesis is true [58]. Also, uniformly distributed *p*-values, with a mean of 0.5 and a variance of 1/12 ≈ 0.0833, produce a linear cumulative distribution function corresponding to the true overall null hypothesis, implying that points in the cumulative *p*-value plot exhibit a straight line. We generated *n* = 5 Monte Carlo permutations of *k* = 50 integers from 1 to *k* inclusive, and calculated the rank sums and the exact *p*-value of the rank sum differences. For this particular set of permutations, the mean of the (_2_^*k*^) = 1, 225 *p*-values was 0.512 and the variance 0.0824. The left panel of Fig. 2c confirms the intuitive notion that the discrete *p*-values are approximately uniformly distributed under *H*_0_. The right panel plots the 1 − *p*-value against the number of *p*-values (i.e., number of hypothesis tests), expressed in terms of proportions. As can be seen, the ensemble of *p*-values in the cumulative plot is close to the diagonal line, as is to be expected when null hypotheses are all true.

### Exact versus approximate critical differences

Table 3 presents the unadjusted and the Bonferroni-adjusted exact and approximate critical differences for 1 × *N* and *N* × *N* comparisons of Friedman rank sums, for *n* = *k* = 5,10,25,50,100, at the familywise error rate of *α*=.05. The values for *CD*_*M*_ were obtained using the R package *mvtnorm* [59], and the other approximate values using standard distributions available in the R *stats* package [55].

**Table 3:** Exact (*CD*) and approximate critical values of differences in rank sums, at the familywise error rate of *α*=.05

*k*	*n*	max(*d*)	Unadjusted		Bonferroni-adjusted						
					*1 × N* comparison			*N × N* comparison			
			*CD*	*CD*_*N*_	*CD*	*CD*_*N*_	*CD*_*M*_	*CD*	*CD*_*N*_	*CD*_*Q*_	*CD* *χ* ^*2*^
5	5	20	11	10	13	13	13	14	15	14	16
	10	40	15	14	18	18	18	20	20	20	22
	25	100	23	22	29	28	28	32	33	31	35
	50	200	32	31	40	40	39	45	45	44	49
	100	400	45	44	57	56	55	64	63	61	69
10	5	45	20	19	27	27	26	30	32	31	40
	10	90	27	27	38	38	37	44	45	43	56
	25	225	43	42	60	60	58	70	70	68	89
	50	450	60	60	85	84	82	99	99	96	125
	100	900	85	84	120	119	115	141	140	136	177
25	5	120	46	46	70	72	69	83	88	86	*141*
	10	240	65	65	100	102	98	121	124	121	199
	25	600	103	102	160	161	154	194	196	191	315
	50	1200	145	145	227	227	218	276	278	270	445
	100	2400	205	204	321	321	308	392	392	381	629
50	5	245	91	91	146	152	145	175	190	185	*376*
	10	490	128	128	210	215	205	258	268	261	*531*
	25	1225	203	203	337	339	323	417	423	412	840
	50	2450	287	286	478	479	457	595	599	582	1188
	100	4900	405	405	677	678	646	844	846	824	1680
100	5	495	180	180	304	320	302	368	406	395	*1019*
	10	990	255	255	441	452	427	548	573	559	*1441*
	25	2475	403	403	708	714	676	891	906	883	2278
	50	4950	569	569	1005	1010	955	1271	1281	1249	3221
	100	9900	805	805	1425	1427	1350	1805	1812	1766	4555

*Note*: The tabled values satisfy the relation *P*(*D* ≥ tabled value) <.05. For presentational purposes, the approximate critical differences were rounded up to the smallest integer that is not less than the calculated value. Italicized figures in the right-most column represent critical differences exceeding the maximum value of *d*, denoted max(*d*), implying that none of the rank sum differences is significant at the *α*=.05 level

The first point to note from Table 3 is that, at the .05 level, the unadjusted normal-approximate critical differences (*CD*_*N*_) are identical to the exact *CD* for almost all *k* and *n*. In the event one chooses not to control the familywise error rate, the underestimation by *CD*_*N*_ amounts to 1 at most, at least for the values of *k* and *n* considered here.

The close correspondence of normal-approximate and exact *CD* deteriorates once the *p*-value threshold for significance is corrected for multiple testing. In 1 × *N* comparisons, the agreement is quite satisfactory as long as *k* is small relative to *n*, but the normal method overestimates the exact critical value if *k* is larger than *n*. The same goes for *N* × *N* comparisons, but worse. As can be seen, the normal approximation generally improves as *n* gets larger, for constant value of *k*, supporting large-sample normal theory. However, the normal method overestimates the exact critical value considerably if *k* is larger than *n*. The disparity is most pronounced if *k* is large and *n* is small. For example, for *k* = 25 and *n* = 5, the exact *CD* is 83, whereas the (rounded) normal approximate critical difference value equals 88. The normal approximation produces larger than exact *p*-values at the tails and larger than exact critical difference values.

The second point to note is that the ordinary normal method – while understating the evidence against the null hypothesis – is, by and large, the most accurate approximate test of the asymptotic variants studied here. The *CD*_*M*_ for *k* − 1 comparisons with a control tends to underestimate the exact *CD*, even if *n* is large, which may lead one to incorrectly reject the null hypothesis. The same goes, but somewhat less so, for all-pairs comparisons with *CD*_*Q*_. The Studentized range critical value is seen to be too liberal in the sense that it underestimates the critical difference value, even for larger values of *n*, and especially if *n* outnumbers *k*. The asymptotic procedure that draws on the chi-squared distribution is seen to perform inadequately overall. As the inferences are suspect, this test statistic is not advocated as a criterion for judging whether differences in Friedman rank sums are significant.

Hence, in general, the normal approximation is overly conservative if *n* is smaller than *k* and the other approximations are too liberal if *n* is larger than *k,* and this holds even for relatively large values of *n*. For many parameter settings the biases are considerable. In any case, they are large enough to imply that if the observed rank sum difference is near to the critical value, the choice between exact and approximate methods can mean the difference between pairs of groups being considered significantly different or not. It is equally important to note that the above results apply to a familywise error rate of *α*=.05. The disparity between exact and asymptotic critical values increases, if the error rate is set to a lower value (e.g., .01). This issue is well visualized in the right panel of the earlier discussed Fig. 2b.

### Type-I error and mid *p*-values

The critical difference values denoted *CD* in Table 3 were obtained by setting the bound on Type-I error at 5%. For the asymptotic approximate methods, with a continuous reference distribution, the maximum probability of rejecting the null when it is in fact true is equal to *α*=.05. An exact test, however, keeps the actual probability of a Type-I error below 5%, as there are only certain *p*-values possible when working with discrete data. Table 4 reports the actual probability of a Type-I error (i.e., exact *p*-value) and the mid *p*-value, for the unadjusted exact *CD* values presented in Table 3 (column 4).

**Table 4:** Exact and mid *p*-values for unadjusted exact *CD* values

*k*	*n*	*p*-value	mid *p*-value	*k*	*n*	*p*-value	mid *p*-value	*k*	*n*	*p*-value	mid *p*-value
5	5	.0326	.0440	10	5	.0397	.0457	25	5	.0494	**.0521**
	10	.0389	.0471		10	.0496	**.0543**		10	.0494	**.0513**
	25	.0437	.0489		25	.0468	.0495		25	.0487	.0498
	50	.0461	.0498		50	.0492	**.0512**		50	.0495	**.0503**
	100	.0465	.0490		100	.0484	.0497		100	.0494	.0499
50	5	.0485	.0498	100	5	.0493	.0500				
	10	.0500	**.0509**		10	.0493	.0497				
	25	.0493	.0498		25	.0496	.0498				
	50	.0493	.0497		50	.0499	**.0501**				
	100	.0497	.0500		100	.0499	.0500				

*Note*: Bold figures indicate mid *p*-values exceeding the nominal level of *α*=.05.

Note that, whereas the alpha level was set at 5%, the actual probability of a Type-I error for the smallest *n* = *k* = 5 is a little above 3%. For larger values of *k* and *n* the ordinary exact test appears only slightly more conservative than the nominal level. Note further that the mid *p*-value minimizes the discrepancy between the exact *p*-value and the significance level. The mid *p*-value occasionally exceeds the nominal level, and still tends to somewhat underrate the nominal in other instances, although necessarily less so than using the exact *p*-value. As can be seen, the difference between exact and mid *p*-value diminishes as *k* and/or *n* increases and the discreteness of the sample distribution diminishes.

We emphasize in this context that the inferential conservativeness associated with exact *p*-values is introduced by testing at a pre-specified alpha level of significance. In practice, it might be preferable to report observed levels of significance rather than testing at a particular cut-off value.

### Normal error and continuity correction

**Fig. 3:**
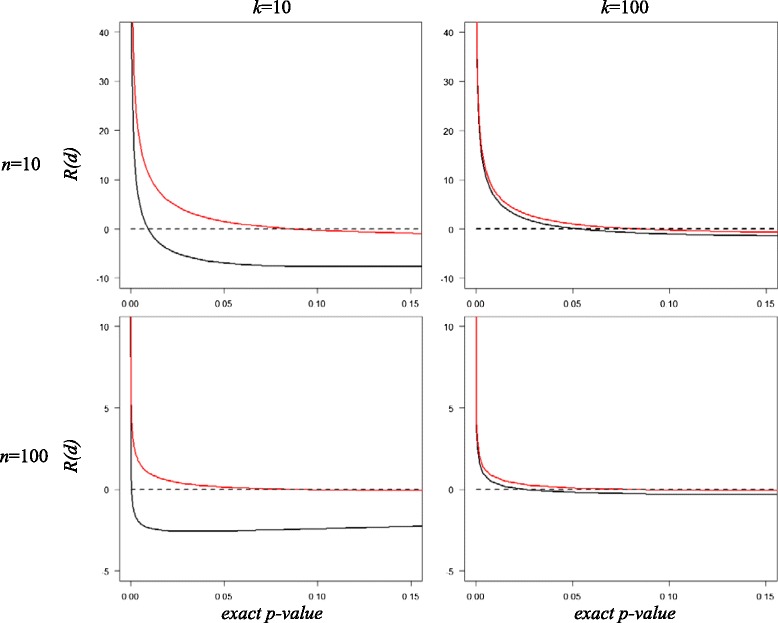
Error normal approximation. Percentage relative error *R*(*d*) of normal approximation with (*red line*) and without (*black line*) continuity correction *versus* exact *p*-value, for *n* = *k* = 10,100

Because the discrete rank sum difference distribution is approximated by a continuous distribution, a correction for continuity is advocated by some (e.g., [24]), to bring the asymptotic probabilities into closer agreement with the exact discrete probabilities. We restrict the discussion to the normal approximation and calculate the percentage relative error of the normal *p*-values to the true *p*-values using

$$ R(d)=100\left\{\frac{P_{\mathrm{normal}}\left( d- c\right)-{P}_{\mathrm{exact}}(d)}{P_{\mathrm{exact}}(d)}\right\}, $$

where *c* is equal to 0.5 or 0 for the normal method with or without continuity correction, respectively. Figure 3 displays the percentage relative error *R*(*d*) versus exact *p*-values, for *n* = *k* = 10,100.

The graphics indicate that the relative error decreases with increasing *n,* both for *k* = 10 and *k* = 100. They also show that, for *k* = 10 and *n* = 10,100, the normal approximation without continuity correction underestimates the true *p*-value if the exact probabilities are large. However, small true *p*-values are overestimated by the normal and this overestimation increases as the probabilities become smaller. Continuity correction brings large normal *p*-values into closer correspondence with the exact *p*-values, but for small *p*-values (i.e., significant results) it may worsen agreement and increase overestimation by the normal. For *k* = 100, the rank sum difference distribution is less discrete and therefore correction for continuity has little effect. This suggests that the neglect of the continuity correction is not a serious matter, and may, indeed, occasionally be an advantage.

Finally, as indicated, the large-sample approximations are derived for the situation where *n* is large. Frequently, however, the number of groups may be quite large whereas the number of replications per group is limited [60]. Such ‘large *k*, small *n*’ situation is fairly common in agricultural screening trials [61] for example, and it also occurs quite often in comparisons of classifiers using ranked data. Published examples in bioinformatics include classifier studies with dimensions *k* = 9 and *n* = 3 [62], *k* = 10 and *n* = 6 [63], and *k* = 13 and *n* = 4 [64]. A similar issue arises in the identification of *k* genes by ranking using *n* different algorithms, for example, *k* = 13 and *n* = 5 as in [65], and *k* = 88 and *n* = 12 as in [66]. Such ‘large *k*, small *n*’ data are common in gene-expression profiling studies [67, 68]. Particularly for these data conditions, the choice of an appropriate test statistic is vitally important to the validity of research inferences.

### Application

We present two data examples to illustrate potential non-equivalence of exact and approximate inference, and the benefit of exact calculation. Recall that we assume that the data are such that it is appropriate to perform the Friedman test. We pass no judgement on this, as that would require expertise in the substantive fields and detailed ‘in-house’ knowledge of selection and measurement procedures. For a proper statistical framework for comparison studies see Boulesteix *et al.* [30]. This review study also shows that real-world applications comparing classifiers are often underpowered. That is, in small-sample settings the differences between the performances of pairs of algorithms are sometimes so variable that one is unable to draw statistically meaningful conclusions.

To illustrate the benefit of exact calculation, Friedman rank data on the comparison of qPCR curve analysis methods were obtained from Ruijter *et al.* [69]. The aim of the comparison of the *k* = 11 methods was to test their performance in terms of the following (*n* = 4) indicators: bias, linearity, precision, and resolution in transcriptional biomarker identification. The null hypothesis is that there is no preferred ranking of the method results per gene for the performance parameters analyzed. The rank scores were obtained by averaging results across a large set of 69 genes in a biomarker data file.

Table 5 displays the Friedman rank sums of the methods and, in the upper top triangle, the absolute values of the differences in rank sums. We obtained the Bonferroni-adjusted normal-approximate *p*-value, Bonferroni-adjusted exact *p*-value, and Studentized range approximate *p*-value for the 55 rank sum differences. The results are presented in the upper bottom, lower bottom, and lower top triangles of the table, respectively.

**Table 5:** Friedman rank data for *k* = 11 methods and *n* = 4 performance indicators (Ruijter *et al*. [69])

Method	Rank sum	Cy0	LinRegPCR	Standard-Cq	PCR-Miner	MAK2	LRE-E100	5PSM	DART	FPLM	LRE-Emax	FPK-PCR
Cy0	7		3	3	10	11	15	25	27	29	31	33
LinRegPCR	10	1		0	7	8	12	22	24	26	28	30
Standard-Cq	10	1	1		7	8	12	22	24	26	28	30
PCR-Miner	17	1	1	1		1	5	15	17	19	21	23
MAK2	18	1	1	1	1		4	14	16	18	20	22
LRE-E100	22	1	1	1	1	1		10	12	14	16	18
5PSM	32	0.423	1	1	1	1	1		2	4	6	8
DART	34	0.220	0.578	0.578	1	1	1	1		2	4	6
FPLM	36	0.110	0.307	0.307	1	1	1	1	1		2	4
LRE-Emax	38	0.052	0.156	0.156	1	1	1	1	1	1		2
FPK-PCR	40	**0.024**	0.076	0.076	0.782	1	1	1	1	1	1	
Cy0			1	1	0.993	0.985	0.883	0.216	0.130	0.073	**0.038**	**0.019**
LinRegPCR		1		1	1	0.999	0.972	0.403	0.271	0.169	0.098	0.053
Standard-Cq		1	1		1	0.999	0.972	0.403	0.271	0.169	0.098	0.053
PCR-Miner		1	1	1		1	1	0.883	0.773	0.631	0.477	0.334
MAK2		1	1	1	1		1	0.923	0.833	0.705	0.554	0.403
LRE-E100		1	1	1	1	1		0.993	0.972	0.923	0.833	0.705
5PSM		0.350	1	1	1	1	1		1	1	1	0.999
DART		0.150	0.514	0.514	1	1	1	1		1	1	1
FPLM		0.057	0.232	0.232	1	1	1	1	1		1	1
LRE-Emax		**0.018**	0.094	0.094	1	1	1	1	1	1		1
FPK-PCR		**0.005**	**0.033**	**0.033**	0.738	1	1	1	1	1	1	

*Note*: The *upper top triangle* displays the rank sum differences, *upper bottom triangle* the Bonferroni-adjusted normal approximate *p*-values, *lower bottom triangle* the Bonferroni-adjusted exact *p*-values, and *lower top triangle* the Studentized range approximate *p*-values. Bold figures indicate *p*-values ≤ .05

Straightforward comparison shows that the approximations are conservative estimates of the true probabilities. That is, the smallest exact *p*-values are considerably smaller than both the normal and the Studentized range approximate *p*-values. According to the normal approximate test there is, at a familywise error rate of .05, no evidence that the methods perform differently, except for Cy0 and FPF-PCR, the pair of methods with the largest difference in rank sums. When applying the Studentized range distribution we find a rank sum difference of *d* = 31 or larger to be significant. The true *p*-values are smaller however, and exact calculation provides evidence that the critical difference value at *α*=.05 is *d* = 30, implying that four pairs of methods perform significantly different. This example illustrates the practical implication of using exact *p*-values in the sense that exact calculation uncovers more significantly different pairs of methods than the asymptotic approximations, and may thus lead to different conclusions.

We were reminded by the reviewers of this paper that the Friedman test assumes that the *n* blocks are independent, so that the measurement in one block has no influence on the measurements in any other block. This leads to questioning the appropriateness of the Friedman test in this application. We do not wish to make any firm judgement about this, other than making the observation that the rank scores presented in the source paper ([69]: Table 2) are strongly related. The same goes for the results of a similar analysis of much the same data by other researchers ([64]: Table 1).

The second illustration concerns exact calculation in incomplete designs. Zagar *et al.* [70] investigated the utility of *k* = 12 data transformation approaches and their predictive accuracy in a systematic evaluation on *n* = 10 cell differentiation datasets from different species (mouse, rat, and human) retrieved from the Gene Expression Omnibus. To compare the predictive accuracy performance of the *k* = 12 methods on the *n* = 10 datasets, they used the Friedman test. Table 6 presents the Friedman ranks obtained by ranking the raw scores presented in Table 1 of Zagar *et al.* [70].

**Table 6:** Friedman rank data for *k* = 12 methods and *n* = 10 cell differentiation datasets (Zagar *et al*. [70])

Method	GDS 2431	GDS 2666	GDS 2667	GDS 2668	GDS 2669	GDS 2671	GDS 2672	GDS 586	GDS 587	GDS 2688	Rank sum excluding GDS2688
MCE-euclid-FC	1	2	1	6	6	1	1	10	8	1	36
PCA-FC	5	1	6	1	1.5	12	8	5.5	1	3	41
PLS-AREA	6.5	8	4	3	4.5	5	6	7.5	3	6	47.5
PCA-AREA	4	6.5	3	2	7	11	7	7.5	2	2	50
MCE-euclid-AREA	3	3.5	2	5	9	3.5	5	11	9	4	51
PLS-FC	9	5	8	4	1.5	3.5	12	5.5	5.5	5	54
SVMRank-FC	11	9	5	8	8	6	3	1	5.5	7	56.5
SVMRank-AREA	9	11	9	7	3	10	2	2	4	8	57
PLS-FC-time	9	3.5	11	11	4.5	8	10	3	10	9	70
PLS-AREA-time	6.5	6.5	12	12	12	9	4	4	7	10	73
Pathrecon	2	12	7	10	11	2	9	9	11		73
PCA-Markers	12	10	10	9	10	7	11	12	12		93
Exact *p*-values for MCE-euclid-FC *vs* PLS-AREA-time											
	*d*	*k*	*n*	*k*_*1*_	*n*_*1*_	*k*_*2*_	*n*_*2*_	unadjusted	Bonferroni-adjusted		
									1 × *N* comparison		*N* × *N* comparison
Excluding GDS2688	37	12	9					**0.016**	0.174		1
Including GDS2688	46			12	9	10	1	**0.003**	**0.038**		0.230

*Note*: Bold figures indicate *p*-values ≤ .05

Note that the ranks of Pathrecon and PCA-Markers for dataset GDS2688 are missing. Zagar *et al.* [70] therefore decided to exclude all ranks within GDS2688 from the computation of the rank sums and restricted their analysis to *n* = 9 datasets. The rank sums excluding GDS2688 are displayed in the right-most column of Table 6.

Instead of deleting GDS2688, the missing data for Pathrecon and PCA-Markers could be dealt with by substitution, for example by imputing the mean of the observed raw scores, followed by re-ranking the 12 methods according to their scores on GDS2688. However, as noted by the authors, the score of PCA-Markers for GDS2688 is not given because “stem cell differentiation markers are not relevant for the process studied in this dataset” ([70]: 2549). Hence the rank score is missing by design, and thus imputation is inappropriate at least for the PCA-Markers method.

An alternative procedure is to divide the *n* = 10 independent ranking into two different parts, one consisting of *k* = 12 methods and *n* = 9 datasets and the other one having *k* = 10 methods and *n* = 1 dataset. The computation of exact *p*-values in such incomplete design is readily accomplished, since the probabilities are easily obtained by the method outlined above. These *p*-values afford the possibility to conduct valid significance tests using all available rank data.

The bottom part of Table 6 presents the exact *p*-values obtained for the comparison of the MCE-euclid-FC and the PLS-AREA-time methods. Additional file 6 has the R code to reproduce the results. The next-to-last row displays the exact *p*-values for the difference *d* = (73–36=)37 in rank sums, if the ranks for GDS2688 are not included in the sums. The bottom row shows the exact *p*-values for the rank sums difference *d* = ([73 + 10]-[36 + 1]=)46 if the two rank sums include the available ranks of the methods for GDS2688. Note that for this particular comparison at least, the latter *p*-values, whether adjusted or not, are considerable smaller than the *p*-values obtained after listwise deletion of missing rank data.

The *p*-value probabilities pertaining to difference of sums of all available rank data can also be estimated using permutation testing and most likely also with methodology such as Laplace approximation or the saddlepoint method. However, these stochastic and deterministic approximations tend to become rather complicated and more cumbersome to work with than the exact computation method described here.

## Conclusions

We provide a combinatorial exact expression for obtaining the probability distribution of the discrete rank sum difference statistic for pairwise comparison of Friedman rank sums. The exact null distribution contributes to the improvement of tests of significance in the comparison of Friedman rank sums, and constitutes a framework for validating theoretical approximations to the true distribution. The numerical evaluations show that, in multiple comparison testing, determining the exact critical difference and the true *p*-value offers a considerable improvement over large-sample approximations in obtaining significance thresholds and achieved levels of significance. The empirical applications discussed exemplify the benefit, in practice, of using exact rather than asymptotic *p*-values.

Of the large-sample approximation methods considered in this study, the simple normal approximation corresponds most closely to the exact results, both for many-one and all-pairs comparisons. However, the difference between exact and normal approximate *p*-values can be large for significant events further in the tail of the distribution. Such events occur, in particular, whenever the number of groups *k* is large and the number of blocks *n* is small. In a multiple testing context with ‘large *k* and small *n*’, application of the normal approximation increases the probability of a Type-II error, hence false acceptance of the null hypothesis of ‘no difference’. The exact *p*-values also greatly improve the ability to detect significant differences if the observed rank sum differences are close to the approximate critical value. In such situation, the choice between exact and approximate methods can mean the difference between pairs (classifiers) being considered significantly different or not. Further, we typically prefer tests that are as accurate as possible while still being fast to compute. As the exact *p*-values can be computed swiftly by the method outlined in this note, there is no longer need to resort to occasionally flawed approximations.

Finally, the rank sum and rank product statistics are widely used in molecular profiling to identify differentially expressed molecules (i.e., genes, transcripts, proteins, metabolites) [67, 68, 71]. Molecule selection by ranking is important because only a limited number of candidate molecules can usually be followed up in the biological downstream analysis for subsequent study. The non-parametric statistic discussed here is potentially an additional new tool in the toolbox of methods for making justified, reproducible decisions about which molecules to consider as significantly differentially expressed.

## Additional files

Additional file 1: Proof of Theorem 1. (PDF 59 kb)
Additional file 2: Proof of Theorem 2. (PDF 51 kb)
Additional file 3: Friedmanrsd. A.zip file providing the script of the algorithm implemented in R. (ZIP 2 kb)
Additional file 4: Numerical example for *k* = 3, *n* = 2. (PDF 67 kb)
Additional file 5: Number of compositions of *d* for *k,n* = 2,…,6. (PDF 65 kb)
Additional file 6: Computation of *p*-values presented in Table 6. A.zip file providing the R code to reproduce the exact *p*-values presented in Table 6. (ZIP 1 kb)

## Abbreviations

CD: Critical difference
LSD: Least significant difference
