# Supplementary material for: Exact p-values for pairwise comparison of Friedman rank sums, with application to comparing classifiers
Source: BMC Bioinformatics. 2017 Jan 25;18:68. doi: 10.1186/s12859-017-1486-2 (PMC5267387; doi:10.1186/s12859-017-1486-2)
Supplement: Additional file 1: — Proof of Theorem 1. (PDF 59 kb) [file 12859_2017_1486_MOESM1_ESM.pdf]

Exact *p*-values for pairwise comparison of Friedman rank sums, with application to comparing classifiers

by Eisinga, Heskes, Pelzer & Te Grotenhuis, *BMC Bioinformatics*, 2017

THEOREM 1: *For n mutually independent integer-valued rankings, each with equally likely rank scores ranging from 1 to k, the exact probability to obtain pairwise difference d for any two rank sums equals*

$$P(D=d;k,n)=\{k(k-1)\}^{-n}W(D=d;k,n),$$

where

$$W(D=d;k,n)=\{k(k-1)\}^{n}\sum_{h=0}^{n}\binom{n}{h}k^{-h}(1-k)^{-n}\sum_{i=0}^{h}\sum_{j=0}^{h}(-1)^{(j-i)}\binom{h}{i}\binom{h}{j}\binom{k(j-i)-d+h-1}{k(j-i)-d-h},$$

is the number of distinct ways a rank sum difference of d can arise, with d having support on $d=[-n(k-1),n(k-1)]$.

Proof: Our approach exploits the observation that the difference among a pair of rank sums is the sum of rank pair differences. Problem-solving tool is the probability generating function

$$f(t)=\sum_{r=1}^{\infty}p_r t^r,$$

where the probabilities $p_r=P(R=r)$ of the integer rank values r are the coefficients of t^r. Denote two distinct ranks as r_i and r_j, with $r_i \neq r_j$. For a single ranking (i.e., one block), the generating function of the sequence of probabilities of the random rank differences is given by the finite power series

$$f(t;k)=\sum_{r_i=1}^{k-1}\sum_{r_j=r_i+1}^{k}\left\{p_{r_i}p_{r_j}t^{(r_i-r_j)}+p_{r_i}p_{r_j}t^{(r_j-r_i)}\right\}.$$

According to the null hypothesis each ranking within a block is equally likely. Consequently, rank r_i has probability $P(R=r_i)=p_{r_i}=k^{-1}$, and rank r_j has probability $P(R=r_j)=p_{r_j}=(k-1)^{-1}$, for $1\le r_i,r_j\le k$. The generating function of the probabilities may therefore be expressed as

$$f(t;k)=\sum_{r_i=1}^{k-1}\sum_{r_j=r_i+1}^{k}\left\{\frac{1}{k(k-1)}t^{(r_i-r_j)}+\frac{1}{k(k-1)}t^{(r_j-r_i)}\right\}=\left\{\frac{1}{k(k-1)}\sum_{r_i=1}^{k}\sum_{r_j=1}^{k}t^{(r_i-r_j)}\right\}-\frac{1}{k-1}.$$

This function can be put in more compact form by summing the geometric progressions:

$$f(t;k) = -\frac{1}{k(k-1)}\left\{\frac{(t-t^k)}{(1-t)}\frac{(t-t^{-k})}{(1-t)} - (k-1)\right\} = \frac{1}{k(1-k)}\frac{t(1-t^k)(1-t^{-k})}{(1-t)^2} - \frac{1}{k-1}.$$

The form of the first equation on the right demonstrates that the generating function is symmetric in t around zero, that is $f(t^{-1};k) = f(t;k)$. From the convolution theorem (e.g., Feller [49]), the probability generating function of the sum of n mutually independent rankings, each assuming the rank differences $\{-(k-1),\ldots,-1,1,\ldots,(k-1)\}$, is given by the nth power of $f(t;k)$:

$$f(t;k,n) = \left\{\frac{1}{k(1-k)}\frac{t(1-t^k)(1-t^{-k})}{(1-t)^2} - \frac{1}{k-1}\right\}^n,$$

which, according to the binomial theorem, can be rewritten as

$$f(t;k,n) = \sum_{h=0}^{n}\binom{n}{h}\left\{\frac{1}{k(1-k)}\right\}^h \left\{\frac{t(1-t^k)(1-t^{-k})}{(1-t)^2}\right\}^h \left\{\frac{1}{1-k}\right\}^{n-h}.$$

Applying the binomial expansions of $(1-t^k)^h$ and $(1-t^{-k})^h$, the power series expansion of $(1-t)^{-2h}$, and the -1 transformation and the upper negation identity transformation of the binomial coefficient, i.e.,

$$\sum_{l=0}^{\infty}\binom{-2h}{l}(-t)^l = \sum_{l=0}^{\infty}(-1)^l\binom{-2h}{l}t^l = \sum_{l=0}^{\infty}\binom{l+2h-1}{l}t^l,$$

the expression becomes

$$f(t;k,n) = \sum_{h=0}^{n}\binom{n}{h}\left\{\frac{1}{k^h(1-k)^n}\right\}t^h\sum_{i=0}^{h}\sum_{j=0}^{h}(-1)^{(j-i)}\binom{h}{i}\binom{h}{j}t^{k(i-j)}\sum_{l=0}^{\infty}\binom{l+2h-1}{l}t^l.$$

To calculate the probability $P(D=d;k,n)$ of rank sum difference d as the coefficient of the power of t, we first collect all terms involving powers of t, and subsequently make the change of variable $h-k(j-i)+l=d$, yielding

$$f(t;k,n)=\sum_{h=0}^{n}\binom{n}{h}\left\{\frac{1}{k^h(1-k)^n}\right\}\sum_{i=0}^{h}\sum_{j=0}^{h}\sum_{l=0}^{\infty}(-1)^{(j-i)}\binom{h}{i}\binom{h}{j}\binom{l+2h-1}{l}t^{h+k(i-j)+l}$$

$$=\sum_{d=-n(k-1)}^{n(k-1)}\left[\sum_{h=0}^{n}\binom{n}{h}\left\{\frac{1}{k^h(1-k)^n}\right\}\sum_{i=0}^{h}\sum_{j=0}^{h}(-1)^{(j-i)}\binom{h}{i}\binom{h}{j}\binom{k(j-i)+d+h-1}{k(j-i)+d-h}\right]t^d,$$

$$=\sum_{d=-n(k-1)}^{n(k-1)}\left[\sum_{h=0}^{n}\binom{n}{h}\left\{\frac{1}{k^h(1-k)^n}\right\}\sum_{i=0}^{h}\sum_{j=0}^{h}(-1)^{(j-i)}\binom{h}{i}\binom{h}{j}\binom{k(j-i)-d+h-1}{k(j-i)-d-h}\right]t^{-d},\quad |t|<1\wedge t\neq 0,$$

where the second equation follows by noting that whenever $h-k(j-i)+l=d$ and $l\geq 0,$ we have $l+2h-1=k(j-i)+d+h-1$ and $k(j-i)+d-h\geq 0,$ and the third equation follows from the property of symmetry of the generating function. The probability of rank sum difference d is therefore

$$P(D=d;k,n)=\{k(k-1)\}^{-n}W(D=d;k,n),$$

where

$$W(D=d;k,n)=\{k(k-1)\}^{n}\sum_{h=0}^{n}\binom{n}{h}k^{-h}(1-k)^{-n}\sum_{i=0}^{h}\sum_{j=0}^{h}(-1)^{(j-i)}\binom{h}{i}\binom{h}{j}\binom{k(j-i)-d+h-1}{k(j-i)-d-h},$$
$$d=-n(k-1),\ldots,n(k-1),$$

represents the number of composition of d into n parts, where each part is restricted to the interval $[-(k-1),k-1]$. Hence $W(D=d;k,n)$ gives the number of different ways a rank sum difference of d can arise, for n mutually independent rankings, each with equally likely integer-valued ranks $\{1,2,\ldots,k\}$. This completes the proof.

The cumulative distribution of rank sum difference d has generating function $f(t;k,n)/(1-t)$ [see, 50-52], and by repeated application of Pascal's identity

$$\binom{m}{r}+\binom{m}{r-1}=\binom{m+1}{r},\qquad \text{for } 1\leq r\leq m+1,$$

the expression for the exact p-value is obtained as

$$P(D\geq d;k,n)=\sum_{h=0}^{n}\binom{n}{h}k^{-h}(1-k)^{-n}\sum_{i=0}^{h}\sum_{j=0}^{h}(-1)^{(j-i)}\binom{h}{i}\binom{h}{j}\binom{k(j-i)-d+h}{k(j-i)-d-h},\quad d=-n(k-1),\ldots,n(k-1).$$

To illustrate the derivations presented above, Additional file 4 offers a small-sized numerical example (k=3, n=2). Additional file 5 tabulates the number of compositions of d for combinations of n=k=2,…,6, for inclusion in the OEIS [53].
