# Supplementary material for: Exact p-values for pairwise comparison of Friedman rank sums, with application to comparing classifiers
Source: BMC Bioinformatics. 2017 Jan 25;18:68. doi: 10.1186/s12859-017-1486-2 (PMC5267387; doi:10.1186/s12859-017-1486-2)
Supplement: Additional file 2: — Proof of Theorem 2. (PDF 51 kb) [file 12859_2017_1486_MOESM2_ESM.pdf]

Exact *p*-values for pairwise comparison of Friedman rank sums, with application to comparing classifiers

by Eisinga, Heskes, Pelzer & Te Grotenhuis, *BMC Bioinformatics*, 2017

THEOREM 2: *For nonnegative integers d and k*

$$\sum_{i=0}^{h}\sum_{j=0}^{h}(-1)^{(j-i)}\binom{h}{i}\binom{h}{j}\binom{k(j-i)-d+h}{k(j-i)-d-h}=\sum_{s=0}^{h}(-1)^{s}\binom{2h}{h+s}\binom{ks-d+h}{ks-d-h}.$$

Proof: Note that, for nonnegative integers *d* and *k*, the right-most binomial coefficient on the left-hand side equals 0, whenever $j < i.$ We set $j - i = s,$ and rewrite the left side as follows

$$\sum_{s=0}^{j}\sum_{j=0}^{h}(-1)^{s}\binom{h}{j-s}\binom{h}{j}\binom{ks-d+h}{ks-d-h}=\sum_{s=0}^{h}(-1)^{s}\binom{ks-d+h}{ks-d-h}\sum_{j=0}^{h}\binom{h}{j}\binom{h}{j-s}.$$

The upper bound of the outer summation over *s* in the first equation is *j*. However, the same summation result is obtained if the upper bound is *h*, as in the second equation, since

$$\binom{h}{j-s}$$

equals 0, whenever $s > j.$ The second equation additionally takes both the alternating sign term and the right-most binomial coefficient of the first equation out of the summation over *j*, as they are conditioned by *s*.

We subsequently simplify the summation

$$\sum_{j=0}^{h}\binom{h}{j}\binom{h}{j-s}$$

to a closed-form expression, using a special case of Vandermonde's convolution identity, stating

$$\sum_{j=0}^{r}\binom{h}{j}\binom{h}{r-j}=\binom{2h}{r}.$$

We find that

$$\sum_{j=0}^{h}\binom{h}{j}\binom{h}{j-s}=\sum_{j=0}^{h}\binom{h}{j}\binom{h}{h+s-j}=\sum_{j=0}^{h+s}\binom{h}{j}\binom{h}{h+s-j}=\binom{2h}{h+s},$$

where the third equation follows by noting that the binomial coefficient with lower index j equals 0, whenever $j > h.$ It is therefore immaterial to the result whether the upper bound of the summation is h or $h + s.$ Inserting the closed-form expression and rearranging yields

$$\sum_{i=0}^{h}\sum_{j=0}^{h}(-1)^{(j-i)}\binom{h}{i}\binom{h}{j}\binom{k(j-i)-d+h}{k(j-i)-d-h}=\sum_{s=0}^{h}(-1)^{s}\binom{2h}{h+s}\binom{ks-d+h}{ks-d-h},$$

as claimed. This completes the proof.

We additionally note that an elegant alternative expression for the summation on the right hand side of Theorem 2 is

$$\sum_{s=0}^{h}(-1)^{s}\binom{2h}{h+s}\binom{ks-d+h}{ks-d-h}=\sum_{s=0}^{h}(-1)^{s}\binom{2h}{h+s}\binom{ks-d+h}{2h},$$

and that the integer sequence

$$(-1)^{s}\binom{2h}{h+s}$$

is a signed version of the elements in the even-numbered rows of Pascal's triangle (see Sloane's [52] series A062344 and A034870). For $k = 2$, the sequence

$$\sum_{s=0}^{h}(-1)^{s}\binom{2h}{h+s}\binom{ks-d+h}{2h}$$

is a signed version of the OEIS [52] triangle A008949.
