# Supplementary material for: Exact p-values for pairwise comparison of Friedman rank sums, with application to comparing classifiers
Source: BMC Bioinformatics. 2017 Jan 25;18:68. doi: 10.1186/s12859-017-1486-2 (PMC5267387; doi:10.1186/s12859-017-1486-2)
Supplement: Additional file 4: — Numerical example for k = 3, n = 2. (PDF 67 kb) [file 12859_2017_1486_MOESM4_ESM.pdf]

Exact *p*-values for pairwise comparison of Friedman rank sums, with application to comparing classifiers

by Eisinga, Heskes, Pelzer & Te Grotenhuis, *BMC Bioinformatics*, 2017

The following is a small numerical example to illustrate the calculation. Suppose we want to obtain the rank sum difference distribution for $k=3$ groups and $n=2$ blocks. Assuming no ties, the ranks are $r=1,2,3,$ and the rank differences are $-2,-1,1,2.$ The permutation distribution assigns equal probabilities to the three ranks, i.e., $p_r = 1/3,$ for $r=1,2,3,$ and equal probabilities to the four pairwise rank differences, i.e., $p_{r_i} p_{r_j} = 1/3 \times 1/2 = 1/6,$ for $i,j=1,2,3,$ with $i \neq j.$ The outcomes are not equally likely however. There are two different ways to obtain rank sum difference value 1, namely 2-1 and 3-2, but there is only one way to obtain a rank sum difference of 2, namely 3-1.

The generating function of the rank sum differences for a single block generates the probabilities as

$$f(t;3) = \frac{1}{6}t^{-2} + \frac{2}{6}t^{-1} + \frac{2}{6}t^{1} + \frac{1}{6}t^{2} = \frac{1}{6}\left(t^{-2} + 2t^{-1} + 2t^{1} + t^{2}\right).$$

The coefficients of the powers of *t* in the first equation on the right are the probabilities assigned to the rank sum differences, indicated by the powers -2,-1,1,2 of *t*. The coefficients of the powers of *t* within brackets in the second equation are the number of different ways to obtain the rank sum difference in question. For example, rank sum difference -1 has 2 configurations (1-2 and 2-3) and the associated probability is 2/6. The probability generating function of the sum of the rank differences for two blocks, assuming independence of the blocks, is the product of the two generation functions, i.e.,

$$f(t;3,2) = \left(\frac{1}{6}t^{-2} + \frac{2}{6}t^{-1} + \frac{2}{6}t^{1} + \frac{1}{6}t^{2}\right)^{2},$$

which is written compactly as

$$f(t;3,2) = \left\{\frac{1}{6}\frac{t(1-t^{3})(1-t^{-3})}{(1-t)^{2}} - \frac{1}{2}\right\}^{2}.$$

Expanding and collecting terms yields the generating function

$$f(t;3,2)=\sum_{d=-4}^{4}\left[\sum_{h=0}^{2}\binom{2}{h}\frac{1}{3^h(-2)^2}\sum_{i=0}^{h}\sum_{j=0}^{h}(-1)^{(i+j)}\binom{h}{i}\binom{h}{j}\binom{3(j-i)-d+h-1}{3(j-i)-d-h}\right]t^{-d}$$

$$=\sum_{d=-4}^{4}\left[\left\{\frac{1}{6}\right\}^2 W(D=d;3,2)\right]t^{-d}$$

$$=\sum_{d=-4}^{4}\left[(P=d;3,2)\right]t^{-d},$$

where we can read the probability $(P=d;3,2)$ as the coefficient of t^{-d}. The probability mass distribution is obtained as

$$P(D=d;3,2)=\sum_{h=1}^{2}\binom{2}{h}(-3)^{(2-h)}\sum_{i=0}^{h}\sum_{j=0}^{h}(-1)^{(i+j)}\binom{h}{i}\binom{h}{j}\binom{3(j-i)-d+h-1}{3(j-i)-d-h},$$

and the cumulative distribution as

$$P(D\geq d;3,2)=\sum_{h=0}^{2}\binom{2}{h}\frac{1}{3^h(-2)^2}\sum_{i=0}^{h}\sum_{j=0}^{h}(-1)^{(i+j)}\binom{h}{i}\binom{h}{j}\binom{3(j-i)-d+h}{3(j-i)-d-h}\qquad \text{for } d=-4,\ldots,4.$$

As explained in the main text, the *p*-value of non-negative *d* may also be obtained from the simplified expression

$$P(D\geq |d|;3,2)=\begin{cases}2\sum_{h=0}^{2}\binom{2}{h}\frac{1}{3^h(-2)^2}\sum_{s=0}^{h}(-1)^s\binom{2h}{h+s}\binom{3s-d+h}{3s-d-h} & \text{for } d=1,\ldots,4\\ 1 & \text{for } d=0.\end{cases}$$

Table S1 summarizes the results for $k=3$ groups and $n=2$ blocks. In this setting, there are a total of 9 possible (positive and negative) values for the differences in rank sums.

Table S1. Rank sum difference distribution for $k = 3$ and $n = 2$

d	-4	-3	-2	-1	0	1	2	3	4
$W(D = d;3,2)$	1	4	4	4	10	4	4	4	1
$W(D \geq d;3,2)$	36	35	31	27	23	13	9	5	1
$P(D = d;3,2)$	1/36	4/36	4/36	4/36	10/36	4/36	4/36	4/36	1/36
$P(D \geq d;3,2)$	1	35/36	31/36	27/36	23/36	13/36	9/36	5/36	1/36
$W(D = \lvert d \rvert;3,2)$					10	8	8	8	2
$W(D \geq \lvert d \rvert;3,2)$					36	26	18	10	2
$P(D = \lvert d \rvert;3,2)$					10/36	8/36	8/36	8/36	2/36
$P(D \geq \lvert d \rvert;3,2)$					1	.722	.500	.278	.056

In practical settings we are testing absolute differences and, therefore, we use only the right tail of the symmetric distribution. It is immediately clear from the figures in the bottom line of the table that an absolute rank sum difference of 4 would be significant at the (unadjusted) .10 level, as $P(D \geq \lvert 4 \rvert;3,2) < .10$, but not at an alpha level of .05. The R code to compute the results presented in the table is included in Additional file 3. The R script `pexactfrsd` computes the exact *p*-value presented in the bottom row of Table S1 and, optionally, all statistics presented in the bottom panel of the table.
