# Supplementary material for: Exact p-values for pairwise comparison of Friedman rank sums, with application to comparing classifiers
Source: BMC Bioinformatics. 2017 Jan 25;18:68. doi: 10.1186/s12859-017-1486-2 (PMC5267387; doi:10.1186/s12859-017-1486-2)
Supplement: Additional file 5: — Number of compositions of d for k,n = 2,…,6. (PDF 65 kb) [file 12859_2017_1486_MOESM5_ESM.pdf]

Exact *p*-values for pairwise comparison of Friedman rank sums, with application to comparing classifiers

by Eisinga, Heskes, Pelzer & Te Grotenhuis, *BMC Bioinformatics*, 2017

The entries in the tables below are the number of compositions of *d*, referred to in the main text as $W(D=d;k,n)$, for $k,n=2,\ldots,6$. The tabulated figures are for the right half of the distribution (i.e., non-negative *d* values) only. The entries for positive *d* (i.e., $d>0$ only) should be doubled if the number of compositions of the absolute *d* values are required.

k=2

			n		
d	2	3	4	5	6
0	2	0	6	0	20
1	0	3	0	10	0
2	1	0	4	0	15
3		1	0	5	0
4			1	0	6
5				1	0
6					1

k=3

			n		
d	2	3	4	5	6
0	10	24	198	880	5380
1	4	36	152	940	4920
2	4	27	136	810	4440
3	4	14	120	600	3832
4	1	12	68	480	2799
5		6	40	312	1980
6		1	24	165	1324
7			8	90	732
8			1	40	366
9				10	172
10				1	60
11					12
12					1

k=4

d	*n* 2	3	4	5	6
0	28	180	2268	23200	260500
1	16	210	2064	23075	252960
2	15	180	1896	21230	236718
3	12	144	1616	18565	211336
4	10	96	1327	15160	180864
5	4	72	936	12003	146040
6	1	44	628	8810	112644
7		21	392	6005	82488
8		6	226	3760	57192
9		1	104	2215	36936
10			36	1182	22260
11			8	545	12456
12			1	200	6400
13				55	2904
14				10	1113
15				1	340
16					78
17					12
18					1

k=5

d	*n* 2	3	4	5	6
0	60	726	14020	246820	4555950
1	40	786	13360	244100	4479924
2	38	711	12580	231050	4282038
3	32	616	11280	211200	3967972
4	25	501	9670	186025	3564561
5	20	366	8000	157194	3105240
6	10	276	6120	128665	2613194
7	4	186	4480	100720	2128368
8	1	111	3105	75380	1674237
9		56	2024	53760	1269836
10		21	1232	36353	926880
11		6	664	23370	648084
12		1	310	14110	433288
13			120	7890	276012
14			36	4005	166716
15			8	1792	94820
16			1	690	50139
17				220	24288
18				55	10582
19				10	4056
20				1	1335
21					364
22					78
23					12
24					1

k=6

			n		
d	2	3	4	5	6
0	110	2136	59634	1592100	43909940
1	80	2241	57904	1576230	43409280
2	77	2088	55388	1515700	42016245
3	68	1883	51216	1420855	39780200
4	56	1632	45821	1297540	36836370
5	44	1347	39688	1153375	33353784
6	35	1036	33384	996220	29526143
7	20	801	26744	838750	25519272
8	10	576	20704	684960	21539496
9	4	384	15408	542250	17737880
10	1	234	10976	415524	14238543
11		126	7456	307525	11129856
12		56	4803	219220	8461490
13		21	2864	150445	6243984
14		6	1564	98860	4465953
15		1	768	61828	3089816
16			330	36520	2062644
17			120	20160	1324488
18			36	10260	814832
19			8	4755	477516
20			1	1972	264804
21				715	137852
22				220	66726
23				55	29700
24				10	12004
25				1	4332
26					1365
27					364
28					78
29					12
30					1
